# Experimental Study on the Quasi-Static Biaxial Compressive Behavior of Miura-Ori Metamaterials

**DOI:** 10.3390/polym18151817

**Published:** 2026-07-25

**Authors:** Xinmei Xiang, Rujin Wang, Jianzhang Huang, Jiale Huang

**Affiliations:** 1 School of Civil Engineering and Transportation, Guangzhou University, Guangzhou 510006, China; xiangxm@gzhu.edu.cn (X.X.);; 2 School of Mechanical and Electrical Engineering, Guangzhou University, Guangzhou 510006, China

**Keywords:** Miura-ori metamaterials, equalbiaxial compression, gradient metamaterials, energy absorption, yield stress

## Abstract

This study investigates the quasi-static equalbiaxial compression behavior of 3D-printed Miura-ori metamaterials incorporating both out-of-plane and in-plane gradient configurations. The Miura-ori structure, composed of tessellated parallelogram units, exhibits pronounced anisotropic behavior due to its unique folding geometry. To assess this behavior, specimens were fabricated using ABS resin and subjected to equalbiaxial compression in three orthogonal directions: the *xy*-direction (in-plane compression), the *yz*-direction (edge-on side loading), and the *xz*-direction (accordion-like profile loading). In the out-of-plane gradient design, the acute angle *ϕ* was varied across layers, significantly influencing both yield stress and specific energy absorption (SEA). Compared with the uniform design, gradient configurations exhibited reduced mechanical performance in the *xy*-direction and *yz*-direction, and enhanced properties in the *xz*-direction. In addition, in-plane (*x*-direction) gradient structures were evaluated under *xy*-, *yz*- and *xz*- direction compression. The results indicate that gradient configuration specimens exhibit significantly lower yield stress and specific energy absorption than uniform structure specimens, with deformation initiating preferentially in regions with smaller acute angles and lower local stiffness. The results highlight the strong influence of geometric gradation and loading direction on the mechanical performance of Miura-ori metamaterials. This work provides new insights into the design and optimization of origami-inspired energy-absorbing structures for use in advanced mechanical, aerospace, and protective engineering applications.

## 1. Introduction

Porous materials found in nature, such as turtle shells [1], moose antlers [2], and vertebral trabeculae [3], exhibit excellent strength-to-weight ratios and have inspired the development of artificial porous structures, including foams [4], lattices [5], honeycombs [6], and origami-based metamaterials [7,8,9,10]. Due to their superior mechanical performance and energy absorption capabilities, these materials have been extensively studied [11,12]. However, much of the existing research has focused primarily on their response to planar uniaxial loading, which may not fully capture their performance under realistic service conditions involving complex, multiaxial stress states. In many real-world scenarios—such as in aerospace applications, vehicular crashes, and blast events—structural components are subjected to simultaneous compressive forces in multiple directions. Therefore, understanding the mechanical behavior of porous and origami-based metamaterials under biaxial compression is essential to predict performance and guide the design of robust energy-absorbing systems.

It is important to note that in practical engineering applications, porous materials are often subjected to complex, multiaxial loading conditions rather than idealized uniaxial loads. Therefore, it is essential to investigate the mechanical behavior of porous materials under multiaxial loading conditions. Li et al. [13] compared hexagonal honeycomb cores under uniaxial and equalbiaxial dynamic compression and found enhanced performance under biaxial loading. In a subsequent study, Li et al. [14] further investigated the biaxial failure characteristics of different honeycomb configurations in terms of deformation modes, plateau stress, energy absorption, and impact response. Liu et al. [15] examined the yield surface evolution of hexagonal honeycombs and proposed a yield point criterion based on equivalent plastic strain increment. For lattice structures, Guo et al. [16] established a print-angle-dependent constitutive model and demonstrated anisotropic behavior under compression, while Sun et al. [17] showed that lateral constraints increase yield stress and energy absorption under biaxial compression. Chen and Mordehai [18] analyzed the yield surfaces of body-centered cubic and octet-truss lattices under multiaxial loading, revealing pronounced anisotropy in the body-centered cubic lattice. For foam materials, Wang et al. [19] investigated the multiaxial yield behavior of Kelvin foams and found that relative density affects the size but not the normalized shape of the yield surface. Su et al. [20] studied recessed honeycomb structures and demonstrated that off-axis angles strongly influence mechanical behavior, resulting in significant anisotropy and tension–compression asymmetry.

Origami metamaterials are a branch of mechanical metamaterials, which form three-dimensional structures by folding a two-dimensional plane in a uniquely designed manner [21]. Different folding patterns can result in origami structures with distinct mechanical properties [22,23,24,25]. Origami has been widely applied in various engineering fields, including deployable space structures [26], origami robots [27,28], architecture and civil engineering [29,30], and energy-absorbing structures [31,32], among others. Xiang et al. [33] reviewed the deformation and energy absorption capabilities of origami structures under static and dynamic loads, with particular focus on the Miura-ori structure. Miura-ori was first proposed by Miura in 1972 [34], and subsequent research has further explored this structure. Zhang et al. [35] conducted quasi-static compression tests on Miura-ori metamaterials and honeycomb structures. The results indicated that the energy absorption capacity of the Miura-ori structure was superior to that of the honeycomb. Xiang et al. [36] compared the compression behavior between a flat plate and a Miura-ori plate, observing that the latter absorbed more energy during deformation. Additionally, Lv et al. [37] conducted quasi-static tests on Miura-ori plates and their corresponding sandwich panels, and the results showed that Miura-ori sandwich panels exhibited superior energy absorption performance.

Previous studies have demonstrated the significant potential of gradient structures for energy absorption. Ma et al. [38] designed a four-layer graded Miura-ori structure that exhibited significantly improved SEA compared with a uniform counterpart. Yuan et al. [39] proposed a Miura-ori metamaterial with in-plane and out-of-plane gradient variations, achieving graded stiffness and enhanced energy absorption, with SEA increasing by up to 68% under out-of-plane quasi-static compression. Xiang et al. [40] reported that graded Miura-ori structures exhibited superior mechanical properties and energy absorption performance compared with uniform designs. Wu et al. [41] developed a sine-based gradient origami structure and found that gradient creases enabled stable progressive folding while providing higher strength and better energy absorption than uniform crease patterns. However, these studies were limited to uniaxial loading conditions. The mechanical response of graded Miura-ori structures under biaxial compression, where stress interaction and load redistribution may significantly affect the deformation and failure mechanisms, has not yet been systematically investigated. Kintscher et al. [42] studied the stiffness and failure characteristics of origami sandwich structures under combined lateral shear and vertical compression loads, finding that the structure’s compression strength and stiffness decreased with increasing initial shear deformation. Sturm et al. [43,44] investigated the combined bending-compression loading of origami sandwich panels and derived failure criteria from a multiscale modeling approach to describe the macroscopic behavior of the folded core under combined loading.

While previous studies have demonstrated that graded Miura-ori structures can improve deformation stability and energy absorption under uniaxial compression, their mechanical response under multiaxial loading conditions remains largely unexplored. In practical engineering applications, origami-based structures are frequently subjected to simultaneous loads along multiple directions. The interaction between geometric gradients and biaxial stress states may fundamentally alter load transfer mechanisms, deformation localization, yield behavior, and energy absorption characteristics. Therefore, it remains unclear whether the beneficial effects of gradient designs observed under uniaxial loading can be retained under biaxial compression. To address this knowledge gap, the present study systematically investigates the quasi-static equalbiaxial compression behavior of graded Miura-ori metamaterials and reveals the influence of loading direction on deformation mechanisms, yield stress evolution, and energy absorption performance.

## 2. Materials and Methods

### 2.1. Geometric Design

**Figure 1:**
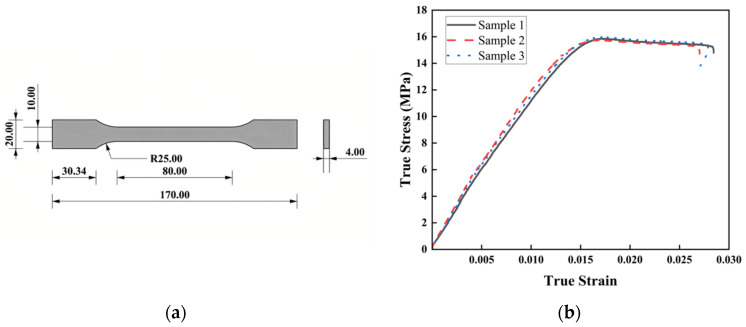
Tensile tests: (**a**) dimensions of the dog-bone specimen (unit: mm) and (**b**) the true stress–true strain curves from the tensile test.

In this study, ABS resin was selected as the base material for the Miura-ori structures. All specimens were fabricated using a Raise3D Pro3Plus 3D printer (Raise3D Technologies, Shanghai, China) based on Fused Feposition Modeling (FDM). Since the FDM process inherently introduces anisotropic mechanical properties due to the layer-by-layer deposition mechanism, the printing orientation, raster pattern, and interlayer bonding quality may influence the mechanical response of the printed structures. Therefore, all specimens were printed with identical build orientation and manufacturing parameters to minimize variability caused by process-induced anisotropy. A summary of the printing parameters used is provided in Table 1. Tensile tests were conducted in accordance with ASTM D638 [45] using a universal testing machine at a crosshead speed of 1 mm/min on 3D-printed dog-bone-shaped specimens. An electronic extensometer was employed to measure the initial small deformation of the specimens. This approach enables the accurate determination of the elastic response and the onset of yielding, while providing a precise zero-strain reference for the reliable evaluation of mechanical properties. Three identical specimens were tested, with each test repeated three times. The specimen geometry is shown in Figure 1a, and the true stress–true strain curves converted from the experimental results are presented in Figure 1b. The basic mechanical properties of the ABS resin are summarized in Table 2.

**Figure 2:**
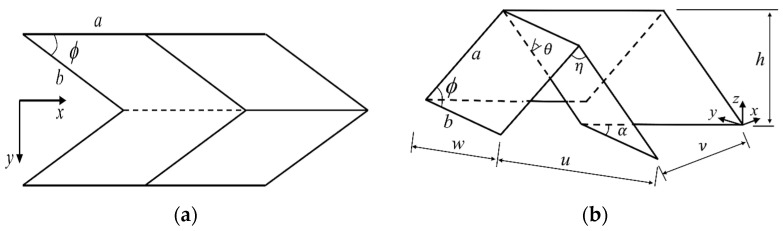
A single Miura-ori unit sketch: (**a**) flat unit and (**b**) folding unit.

The Miura-ori structure used in this work is a rigid origami configuration composed of periodically repeating unit cells, as illustrated in Figure 2a. Each unit cell consists of four identical parallelogram facets arranged in a tessellated folding pattern. The mountain and valley folds are denoted by solid and dashed lines, respectively. The folded geometry shown in Figure 2b results from crease-based transformations applied to the flat sheet in Figure 2a. The edges of the parallelograms are labeled as *a* and *b*, with the acute angle between the two edges denoted as ϕ. The dihedral angle θ, edge angle η, and angle α are used to determine the folding configuration. The overall dimensions of the origami unit cell are represented by parameters such as *h*, *w*, *u*, and *v*. The geometric relationships between these parameters follow the formulations presented in prior studies [46], and are expressed as:

(1) $$\cos\alpha =\sin^{2}\phi \cos\theta +\cos^{2}\phi$$

(2) $$\cos\eta =\frac{\sin^{2}\phi \cos^{2}(\frac{\theta }{2})-\cos^{2}\phi }{\sin^{2}\phi \cos^{2}(\frac{\theta }{2})+\cos^{2}\phi }$$

(3) $$u=2a\cdot \sin\frac{\eta }{2}$$

(4) w=b⋅cosα

(5) $$v=2b\cdot \sin\frac{a}{2}$$

(6) $$h=a\cdot \cos\frac{\eta }{2}$$

where $\eta \in \left[0,180{}^{\circ}\right]$, $\phi \in \left[0,90{}^{\circ}\right]$, $\alpha \in \left[0,2\phi \right]$, and $\theta \in \left[0,90{}^{\circ}\right]$.

**Figure 3:**
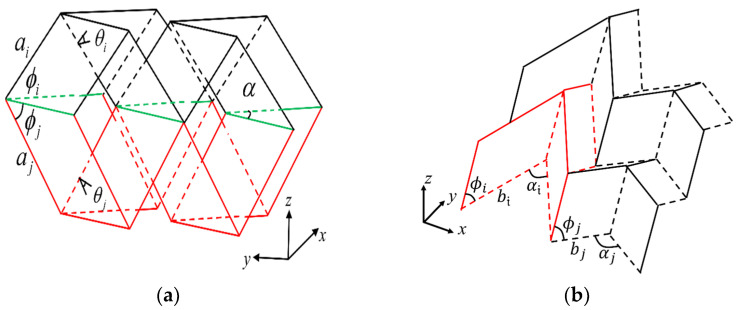
Sketch of graded models: (**a**) model with the gradient in the *z*-direction; (**b**) model with the gradient in the *x*-direction.

When Miura-ori units are stacked in the out-of-plane direction (*z*-direction) (see Figure 3a), certain parameters need to be constrained to ensure a perfect connection between layers. For each layer, the dimensions *w*, *u*, *v*, and the crease angle α of the unit cell in Figure 2b should remain unchanged. In the out-of-plane direction (*z*-direction), the edge length *a*, dihedral angle *θ*, and crease angle *η* vary with the acute angle *ϕ* for the multilayer gradient metamaterial. The relationships between these parameters are as follows:

(7) $$b_{j}=b_{i}$$

(8) $$a_{j}=a_{i}\frac{\cos\phi_{i}}{\cos\phi_{j}}$$

(9) $$\theta_{j}=\arccos\left(\frac{\sin^{2}\phi_{j}-2\sin^{2}\left(\frac{\theta_{i}}{2}\right)\sin^{2}\phi_{i}}{\sin^{2}\phi_{j}}\right)$$

When performing gradient design in the in-plane *x*-direction (see Figure 3b), the edge length *a* of two adjacent origami units remains the same, and the two connected origami units have the same crease angle *η*, while the dimension *v* of the unit cell remains unchanged. The edge length *b* and crease angle *α* vary with the acute angle ϕ.

The overall dimensions of the model are specified as follows:

(10) $$L_{x}=n\cdot v$$

(11) $$L_{y}=m\cdot w+b\cdot \cos\frac{a}{2}$$

(12) $$L_{z}=\sum h_{i}=\sum a_{i}\cdot \cos\frac{\eta }{2}$$

where *n* and *m* refer to the unit numbers in the *x* and *y* directions, respectively.

This study investigates two categories of gradient Miura-ori metamaterial structures: one incorporating in-plane gradient variations along the *x*-direction, and the other featuring out-of-plane gradient variations along the *z*-direction. These gradient configurations are designed to systematically examine the influence of geometric non-uniformity on the mechanical behavior under biaxial loading. Uniform Miura-ori metamaterial models Z-1 and X-1 were designed for the out-of-plane *z*-direction and the in-plane *x*-direction, respectively, and their unit cell geometric parameters are presented in Table 3. The geometric parameters of the gradient models for the out-of-plane *z*-direction and the in-plane *x*-direction are shown in Table 4 and Table 5, respectively.

### 2.2. Experimental Setup

**Figure 4:**
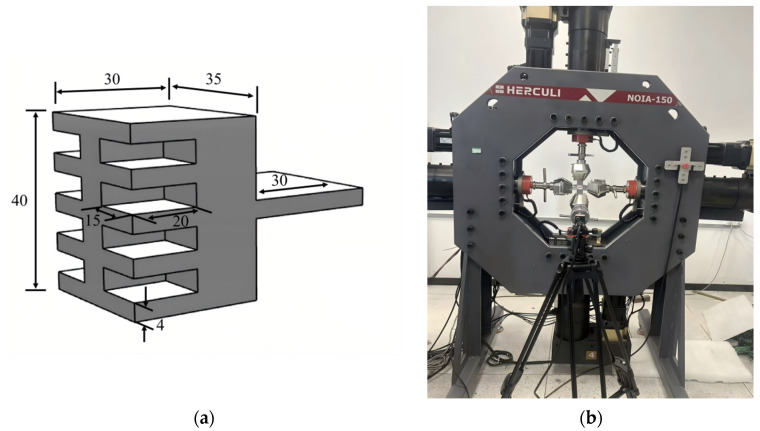
Quasi-static biaxial compression test: (**a**) bracket with two comb-like side modules and (**b**) biaxial compression testing setup.

Figure 4 shows the experimental setup for the equibiaxial compression test. The apparatus consists of two sets of actuators that can independently apply loads along two orthogonal directions, as illustrated in Figure 4b. The system employs four independently controlled motors to drive the actuators, with a load cell mounted on each actuator arm to measure the applied force. In addition, each actuator arm is equipped with an individual displacement sensor. Data synchronization among the four loading axes is ensured through computer-programmed control, enabling coordinated and accurate operation of the system. The original clamps of the experimental setup were designed for biaxial tension tests and thus cannot exert uniform compressive loads. In particular, the Miura-ori structure in this study undergoes large deformation until the entire specimen is fully compacted. Accordingly, custom dedicated clamps are required to connect the Miura-ori structure to the actuator fixtures. As schematically shown in Figure 4a, a novel clamp composed of two comb-shaped side assemblies and surrounding metal pressure plates was designed, enabling mutual sliding between two adjacent clamps. The fabricated clamps were machined from aluminum alloy via CNC milling. Prior to the compression test, a small preload was applied to fix each specimen. A comparison between the results obtained with and without the application of a preload indicates that the preload does not affect the initial stiffness of the structure. All four clamp indenters advanced synchronously at a constant rate of 0.6 mm/min to impose equibiaxial compression on the sample. To ensure data reliability and reproducibility, a minimum of three repeated trials were conducted for each loading scenario. The stress–strain responses exhibited good consistency, and the standard deviations of the yield stress, EA and SEA were generally below 3%. This high repeatability confirms that the quantitative improvements reported in this study are statistically significant and not artifacts of individual specimen defects. The results presented in the following sections represent the average response of these valid tests.

### 2.3. Finite Element Modelling and Experimental Validation

**Figure 5:**
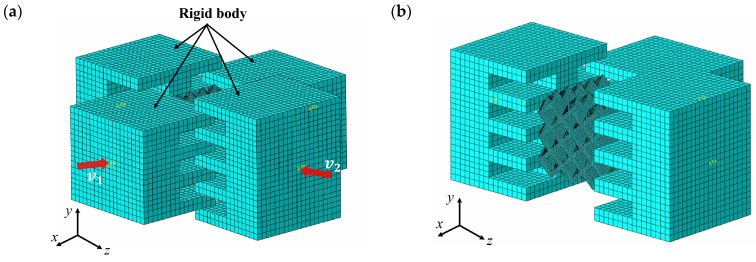
The finite element meshed model: (**a**) fixtures, and (**b**) specimen in the fixtures.

Quasi-static compression simulations were conducted using the explicit solver Abaqus/Explicit. The finite element model is shown in Figure 5. All fixture components were defined as rigid bodies. All degrees of freedom of the fixtures, except those along the loading direction, were fully constrained, and a constant velocity boundary condition of 0.12 m/s was applied in the unconstrained direction. In the contact definition, the tangential behavior was modeled using a penalty formulation with a friction coefficient of 0.15, while the normal behavior was defined as hard contact. A general contact algorithm was employed to account for all possible surface interactions. Based on the convergence study, the mesh size of the Miura-ori structure was set to 0.25 mm. Both the Miura-ori structure and the fixtures were discretized using eight-node reduced-integration solid elements (C3D8R). During compression, the ratio of kinetic energy to internal energy remained below 5%, indicating negligible inertial effects. Therefore, the loading condition can be regarded as quasi-static at the applied loading rate.

**Figure 6:**
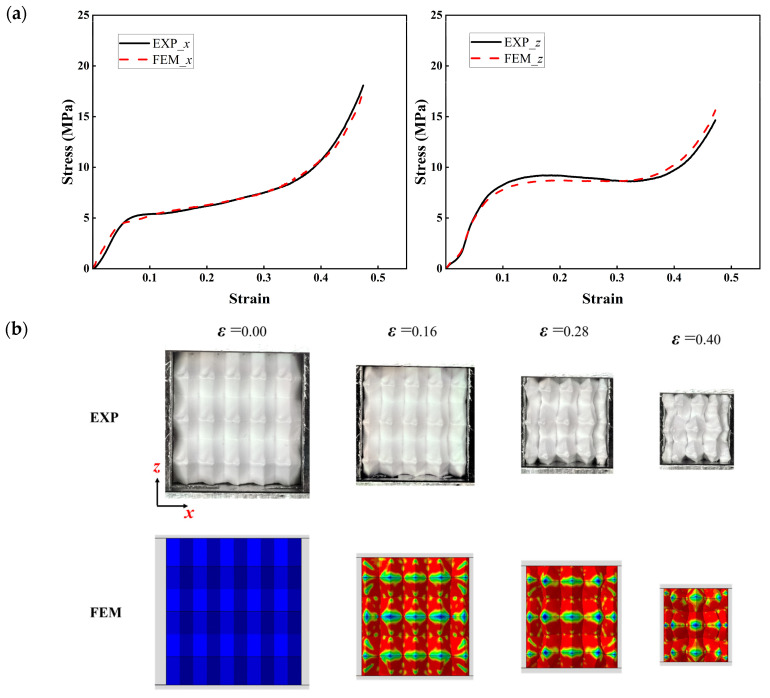
Comparison of experimental and numerical quasi-static equibiaxial compression responses of model Z-1 under *xz*-direction loading: (**a**) stress–strain curve and (**b**) deformation process.

The stress–strain responses of model Z-1 under *xz*-direction loading obtained from both experiments and FE simulations are shown in Figure 6a. The numerical results exhibit good agreement with the experimental curves throughout the entire deformation process. In terms of deformation behavior, Figure 6b compares the structural configurations from experiments and simulations at different strain stages. The FE model successfully reproduces the progressive crushing mechanism observed in the experiments, accurately capturing local buckling and the sequential collapse of individual units. The excellent agreement in deformation modes between experiments and simulations validates the reliability of the numerical model in representing the compression behavior of the Miura-ori structure.

## 3. Results and Discussions

### 3.1. Graded Design in the z-Direction

Previous studies have demonstrated that stacked Miura-ori structures exhibit anisotropic mechanical properties, with deformation modes varying significantly along the *x*, *y*, and *z* directions [40]. To investigate these directional dependencies, this section presents a series of equalbiaxial compression tests performed on Miura-ori metamaterials with uniform and out-of-plane gradient configurations. The specimens are tested in three orthogonal loading directions (*xy*, *yz*, and *xz*) to evaluate how geometric gradation in the *z*-direction influences deformation patterns and mechanical behavior.

#### 3.1.1. Deformation Process

In this study, although the anisotropic mechanical behavior of FDM-printed ABS may influence local deformations and stress distributions within individual panels of the Miura-ori structure, the overall response under biaxial compression is dominated by the geometric folding pattern of the Miura-ori. The minor variations caused by anisotropy may affect local deformations or initial buckling but do not alter the main conclusions of this work.

**Figure 7:**
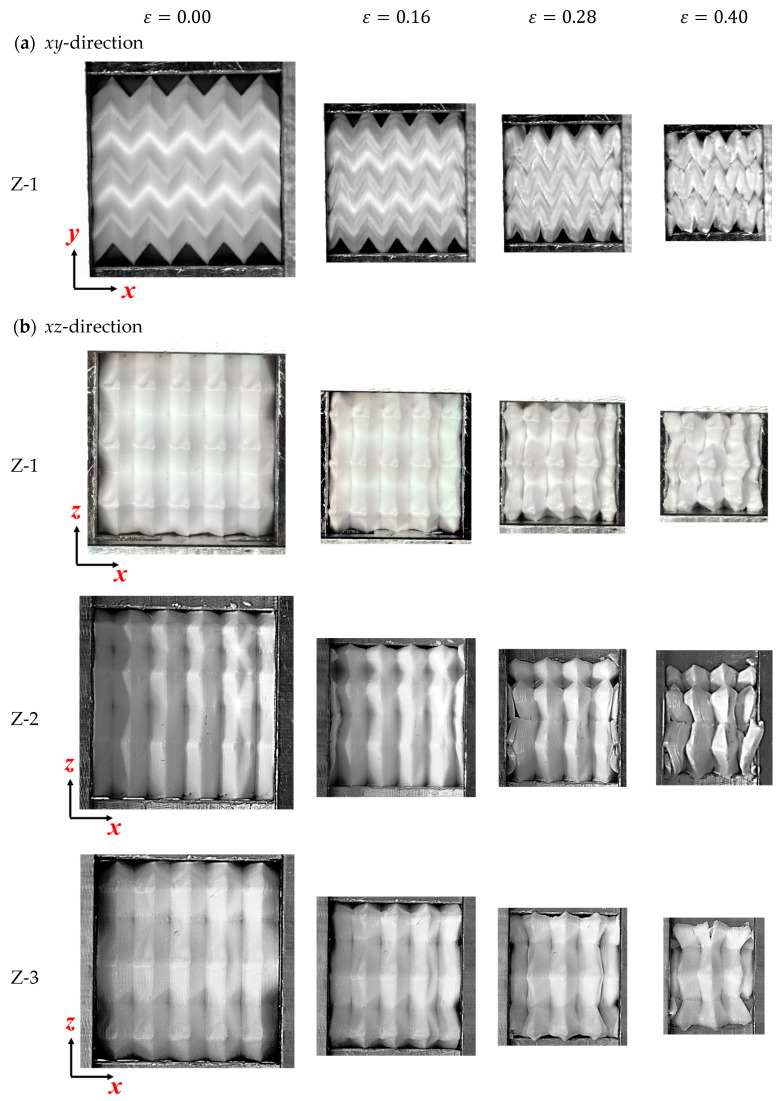

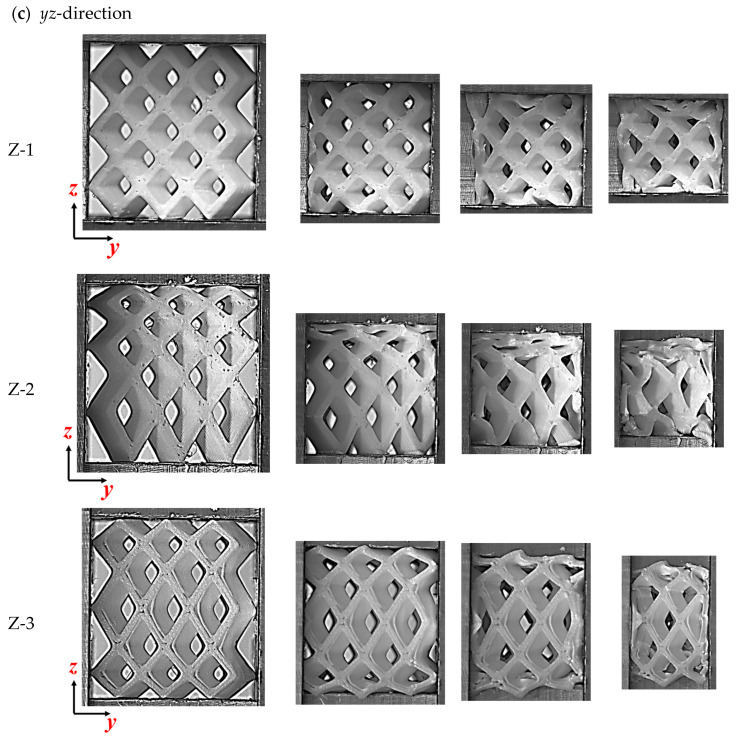
Deformation process: (**a**) *xy*-direction, (**b**) *xz*-direction, and (**c**) *yz*-direction.

The evolution of proportional biaxial compressive deformation of different models in the out-of-plane direction under various orientations is presented in Figure 7. The strain defined in the figure refers to the strain in the *x*-direction for the *xy*-direction and *xz*-direction, and the strain in the *z*-direction for the *yz*-direction. Figure 7a presents the structural response of Model Z-1 under compressive loading in the xy-direction. Experimental observations indicate that all layers undergo synchronous and cooperative deformation under biaxial compression, while boundary cells in contact with the rigid constraint interface deform preferentially. This preferential boundary deformation is likely associated with the interaction between the specimen and the loading platens, which may alter the local stress distribution near the boundaries. The deformation in the x-direction is dominated by rotation around the crease angle α, whereas deformation in the y-direction manifests primarily as contraction along the crease lines. Local cracks initiate when the compressive strain reaches 0.28, with fracture occurring at the structural creases during densification. This behavior can be explained by the rotational kinematics of the Miura-ori facets, where deformation concentrates at the crease lines due to geometric discontinuities. Stress accumulation at the crease lines, enhanced by rotational motion, leads to crack initiation and eventual fracture upon densification. Comparative analysis shows that the deformation sequences of different gradient models in the *xy*-direction are highly consistent with that of Model Z-1, demonstrating that the geometric parameter gradient design exerts a weak regulatory effect on the deformation mode within the *xy*-direction.

Figure 7b illustrates the deformation evolution of different models under compressive loading in the *xz*-direction. The deformation modes of the three models in the *x*-direction are consistent with those under *xy*-direction loading, while remarkable differences are observed in the *z*-direction. Specifically, the uniformly designed Model Z-1 exhibits a typical uniform deformation characteristic, with all layers deforming simultaneously. This uniform deformation arises from the identical stiffness of all origami layers in the z-direction, which ensures that each layer bears equal compressive load and enters the plastic deformation stage at the same time. In contrast, Model Z-2 adopts a gradient angle design strategy, in which the acute angle increases monotonically along the positive *z*-direction from top to bottom. Its deformation mode strictly follows the gradient distribution law, namely that layers with smaller acute angles preferentially enter the plastic deformation stage. Notably, Model Z-3 adopts a bidirectional gradient design, where the acute angle increases symmetrically from the upper and lower edges toward the middle layer. Experimental results reveal that the deformation mode of this structure is strongly correlated with the spatial distribution of angles, presenting a typical deformation propagation characteristic from the edges to the center. Overall, Miura-ori layers with smaller acute angles ϕ are more susceptible to deformation. According to Equation (1), as the acute angle ϕ decreases, the fold angle η increases, which reduces the structural stiffness of the origami layer and consequently leads to a decrease in its load-bearing capacity during quasi-static compression. The observed fracture in Models Z-2 and Z-3 is attributed to stress concentration at the crease lines in regions experiencing early plastic deformation. In contrast, the uniform Model Z-1 remains intact, as the homogeneous stiffness distribution reduces the tendency for localized deformation and stress concentration. The deformation evolution of different models under compression in the *yz*-direction is shown in Figure 7c. The deformation modes in these two directions are similar to those under *xy*-direction and *xz*-direction loading conditions.

#### 3.1.2. Biaxial Stress–Strain Response

In conventional uniaxial compression tests using a general universal testing machine, only one rigid platen moves to compress the specimen while the other remains stationary. In contrast, the four clamps mounted on the biaxial testing machine in this study move simultaneously to compress the specimen. Accordingly, the load-displacement data acquired from the biaxial testing machine are converted into engineering stress–strain curves, which are defined as follows:

(13) $$\sigma =\frac{F}{A_{0}}$$

(14) $$\varepsilon =\frac{2l}{L_{0}}$$

where *F* represents the compressive load, *A*_0_ denotes the cross-sectional area, *L*_0_ is the original side length of the specimen, and *l* represents the displacement of a single fixed support, which is used to evaluate the shortening range of the Miura-ori metamaterial.

**Figure 8:**
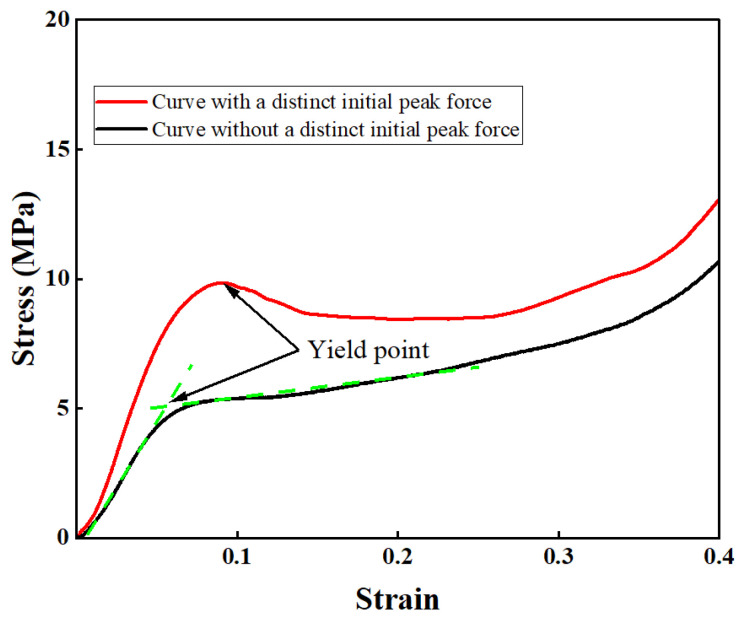
Schematic illustration of the yield stress determination method.

The yield stress was determined based on a consistent criterion derived from the literature [47]. For stress–strain curves exhibiting a distinct local maximum prior to densification, the yield stress was defined as the first peak stress. For curves without a clear peak, yield strength was the intersection point of the tangents of the elastic regime and plastic regime of stress–strain curves. This dual-criterion approach has been widely adopted in previous studies and was applied consistently to all specimens in this work to ensure comparability. The method used to determine the yield stress in this study is illustrated in Figure 8.

**Figure 9:**
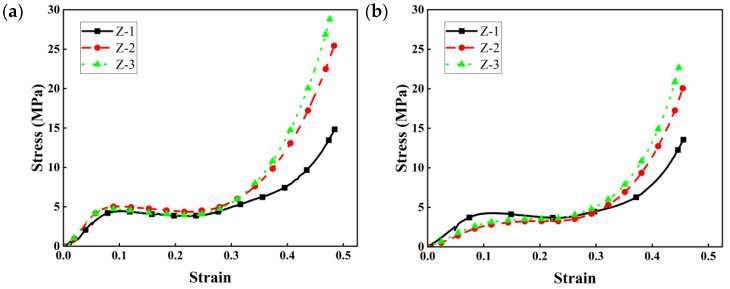
Stress–strain curve under equalbiaxial compression in the *xy*-direction: (**a**) *x*-direction and (**b**) *y*-direction.

**Figure 10:**
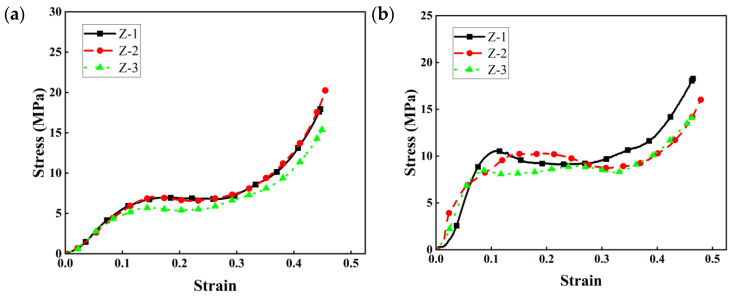
Stress–strain curve under equalbiaxial compression in the *yz*-direction: (**a**) *y*-direction, and (**b**) *z*-direction.

**Figure 11:**
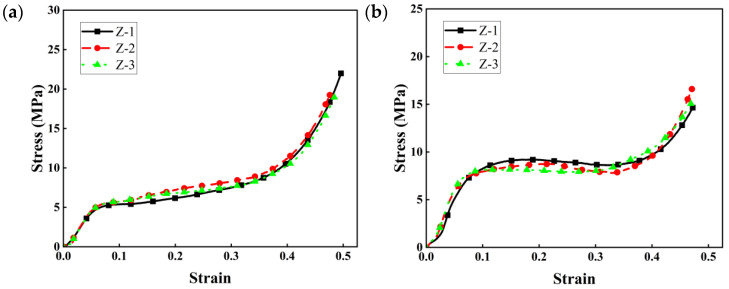
Stress–strain curve under equalbiaxial compression in the *xz*-direction: (**a**) *x*-direction and (**b**) *z*-direction.

Figure 9, Figure 10 and Figure 11 show the stress–strain curves for equibiaxial compression in different directions. It is observed that the stress–strain curves of all models under equibiaxial compression along various directions can be roughly divided into three stages. The first is the elastic stage. For all models under different directional combinations, the elastic modulus in every single direction is nearly identical, and the stress rapidly reaches the yield point at a small strain within this stage. Subsequently, the specimen enters the plastic stage, where an obvious plateau region can be identified for all models under compression with different directional combinations. The evolutionary trend and plateau stress in the plateau region differ among directional combinations and models. In the xy-plane and xz-plane, the evolutionary trend and plateau stress of each direction for all models are almost equivalent. This indicates that the angular gradient design in the out-of-plane direction exerts no significant effect on the load-bearing capacity under compressive loading in the xy and xz directions. In the yz-plane, Model Z-1 and Model Z-2 possess the same plateau stress in the y-direction, both of which are higher than that of Model Z-3. In the z-direction, distinct trends are observed for different models: Model Z-1 presents a trend of initial decrease followed by an increase; Model Z-2 shows a pattern of an initial peak followed by a trough; Model Z-3 exhibits a variation trend of trough–peak–trough. Finally, the specimen enters the densification stage, in which the compressive force rises sharply, and the specimen reaches a fully densified state.

**Figure 12:**
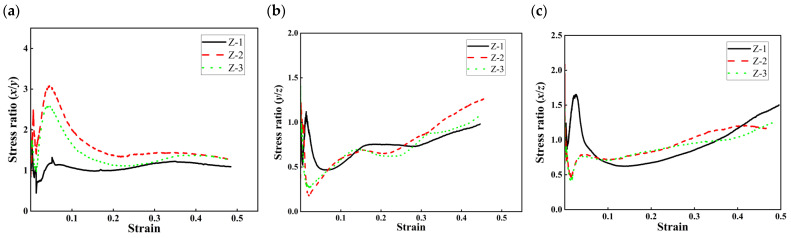
Stress ratio curves under equalbiaxial compression in different directions: (**a**) *xy*-direction, (**b**) *yz*-direction, and (**c**) *xz*-direction.

Figure 12 presents the stress ratio relationships under equibiaxial compression in different directions. The stress ratio is defined as ($R_{ij}=\sigma_{i}/\sigma_{j}$), where ($R_{ij}$ > 1) indicates that the (i)-direction carries a larger stress contribution than the (j)-direction under equal biaxial strain, while ($R_{ij}$ < 1) indicates that the (j)-direction dominates. Therefore, this parameter is used here as a quantitative indicator of directional stress transfer and stiffness anisotropy. In the *xy*-direction, the stress ratio (*x*/*y*) of the three models is always greater than 1, indicating that the stiffness in the *x*-direction is higher during compression and that a larger portion of the load is carried in the *x*-direction. Previous studies have shown that the crushing force in the *y*-direction is larger than that in the *x*-direction under uniaxial compression [40], which demonstrates that the mechanical performance of the Miura-ori structure changes under biaxial compression. The stress ratio curves of all models exhibit an initial fluctuation at small strains, followed by a continuous increase until the strain reaches approximately 0.05, after which the stress ratio gradually decreases and eventually stabilizes. It should be noted that measurements obtained at very small strains and low loads may be influenced by specimen positioning, initial contact conditions, and system compliance. Therefore, the mechanical interpretation is focused primarily on the subsequent stable deformation stage. In this stage, the stress ratio remains above 1 and finally stabilizes at approximately 1.1–1.4, confirming that the *x*-direction maintains a higher stress contribution than the *y*-direction during compression. In addition, the peak stress ratio increases from about 1.2 for Z-1 to approximately 3.0 and 2.6 for Z-2 and Z-3, respectively, indicating that the out-of-plane gradient increases the early-stage stress imbalance between the *x*- and *y*-directions.

In the *yz*-direction, the stress ratio remains consistently lower than 1, suggesting a higher stiffness in the *z*-direction during the compression process, which is consistent with the mechanical behavior under uniaxial compression. Similar to the *xy*-direction, a slight fluctuation is observed in the low-strain region, which may be partially associated with experimental factors. As deformation progresses, all stress ratio curves increase steadily and gradually approach 1; for Z-2, the ratio exceeds 1 at larger strains. This indicates that the dominant stress contribution gradually shifts from the *z*-direction toward a more balanced stress state during later deformation.

In the *xz*-direction, the stress ratio trends differ between the uniform Model Z-1 and the gradient models. For Model Z-1, the stress ratio first rises from 1 to approximately 1.6 and then decreases continuously. By contrast, the stress ratios of all gradient models remain below 1 throughout the compression process, indicating that the z-direction exhibits a stronger resistance to deformation than the *x*-direction. These results further demonstrate that the introduction of gradient configurations significantly alters the load distribution and deformation behavior under biaxial compression.

**Figure 13:**
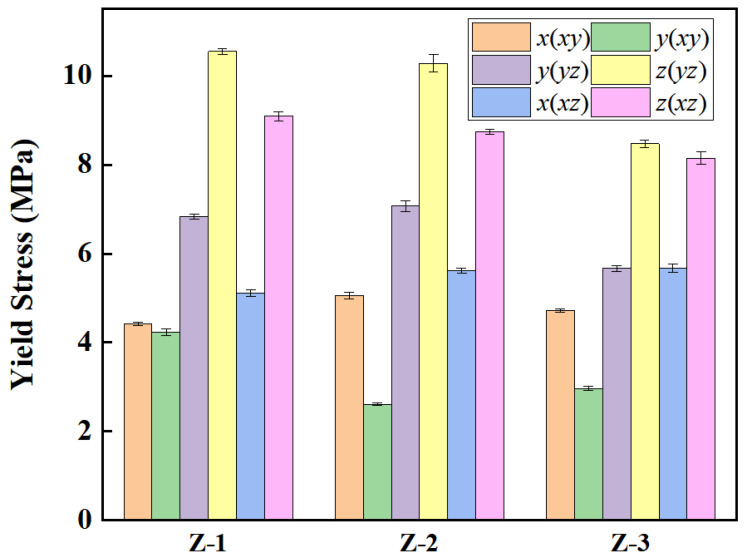
Comparison of yield stress under equalbiaxial compression in different directions for *z*-direction graded structures.

The yield stress in the linear region of the stress–strain response was measured and used to investigate the yield characteristics of Miura-ori metamaterials under equibiaxial compression along different directions. As shown in Figure 13, under *xy*-direction equibiaxial compression, the yield stress of the gradient models in the *x*-direction was higher than that of the uniform models, with increases of 6.8% and 14.3%, respectively; in contrast, the yield stress in the *y*-direction decreased by 29.8% and 38.3%. Under *xz*-direction equibiaxial compression, the gradient models also exhibited higher *x*-direction yield stress than the uniform models, with increases of approximately 10.0% and 11.2%, while the *z*-direction yield stress showed the opposite trend, decreasing by 3.9% and 10.3%. By comparison, under *yz*-direction equibiaxial compression, the yield stress of all gradient models was lower than that of the uniform model. Notably, the maximum reduction in the *z*-direction yield stress was 19.7%, and in the *y*-direction, 17.1%.

By comparing the yield stress of the same model under equibiaxial compression in different directions, it is found that both the uniform and gradient models exhibit the maximum overall average yield stress under *yz*-direction compression. Comparing the *x*-direction yield stress under *xy*-direction and *xz*-direction equibiaxial compression, as well as the *y*-direction yield stress under *xy*-direction and *yz*-direction equibiaxial compression, the yield stresses corresponding to *xz*-direction and *yz*-direction compression are both higher than those under *xy*-direction compression. Further comparison of the *z*-direction yield stress under *yz*-direction and *xz*-direction equibiaxial compression shows that the yield stress under *yz*-direction compression is greater than that under *xz*-direction compression.

**Figure 14:**
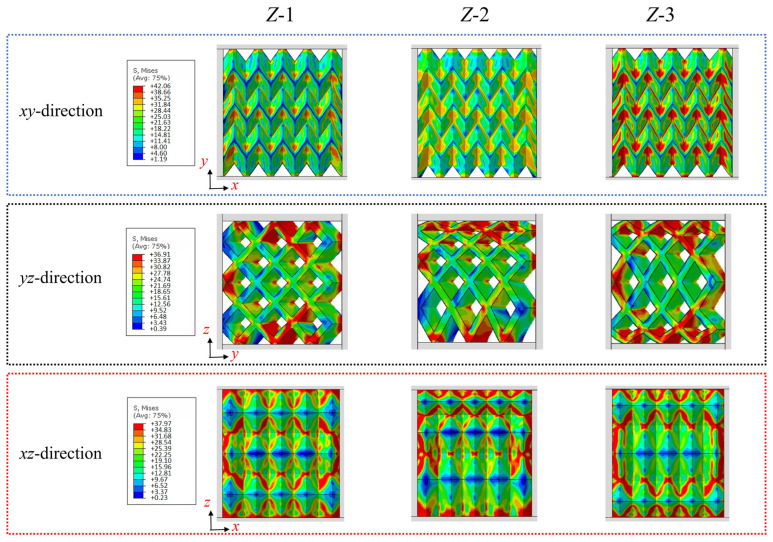
Von Mises stress distributions at the onset of yielding under equibiaxial compression in different directions.

To provide further insight into the yield behavior, the von Mises stress distributions at the onset of yielding obtained from finite element simulations are shown in Figure 14. It can be observed that the introduction of the acute-angle gradient modifies the stress distribution within the structure. Under *xy*-direction compression, the gradient models exhibit a larger area of elevated stress than the uniform model, which is consistent with their higher yield stress in the x-direction. Under *yz*-direction compression, more pronounced local stress concentrations can be observed in the gradient models, corresponding to the reduction in yield stress observed experimentally. A similar trend is found under *xz*-direction compression, where the stress distribution differs noticeably from that of the uniform model. These results indicate that the gradient design influences the yield behavior by altering the internal stress distribution and stress concentration characteristics of the Miura-ori metamaterial.

The following performance indicators are used to evaluate the energy absorption performance of Miura-ori metamaterials: energy absorption (EA) and SEA, which is expressed as follows:

(15) $$EA=\int_{0}^{\varepsilon }d\sigma (\varepsilon )d\varepsilon$$

(16) $$SEA=\frac{EA}{\rho \bar{\rho }}$$

where σ(ε) is the magnitude of the stress generated during the compression test, $\varepsilon_{d}$ is the densification strain point, ρ is the density of the base material, and $\bar{\rho }$ is the relative density of the structure.

**Figure 15:**
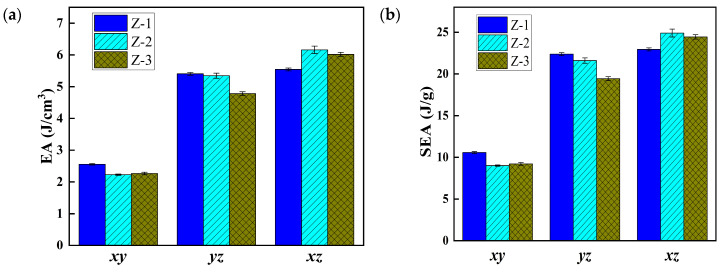
Comparison of EA and SEA for different *z*-direction gradient models under equal biaxial compression in different directions: (**a**) EA and (**b**) SEA.

The comparison of specific energy absorption among different variable-angle gradient models is presented in Figure 15. It can be observed that, under equibiaxial compression in the *xy*-direction, compared with the uniform Model Z-1, the EA of gradient Models Z-2 and Z-3 decreases by 12.87% and 11.38%, respectively, while the SEA decreases by 16.91% and 11.92%, respectively. In the *yz*-direction, relative to Model Z-1, the EA of Models Z-2 and Z-3 is reduced by 1.13% and 11.52%, and the SEA is reduced by 5.71% and 12.04%, respectively. In the *xz*-direction, compared with Model Z-1, the EA of gradient Models Z-2 and Z-3 increases by 11.12% and 8.52%, and the SEA increases by 8.56% and 6.52%, respectively. Compared with previous studies under uniaxial compression, several distinct mechanical characteristics are observed under biaxial loading. Although out-of-plane gradient Miura-ori structures generally exhibit superior energy absorption performance under uniaxial compression [38], the present results demonstrate that under biaxial loading conditions, the gradient configuration may lead to either an enhancement or a reduction in SEA, depending on the loading direction. By comparing the energy absorption of the same model under equibiaxial compression in different directions, both the uniform model and gradient models show a consistent trend, following the order: ${EA}_{xz}>{EA}_{yz}>{EA}_{xy}$.

### 3.2. Graded Design in the x-Direction

#### 3.2.1. Deformation Process

**Figure 16:**
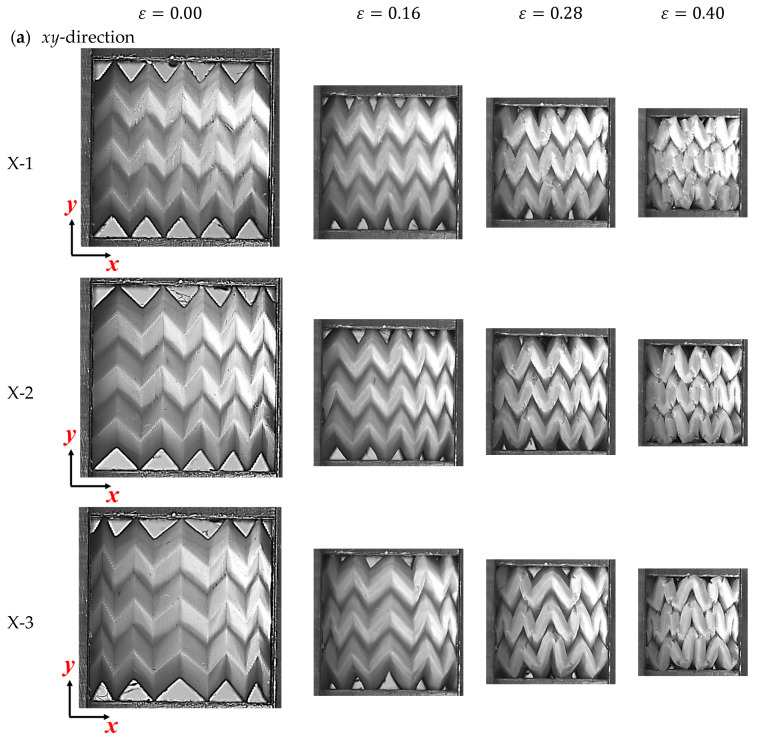

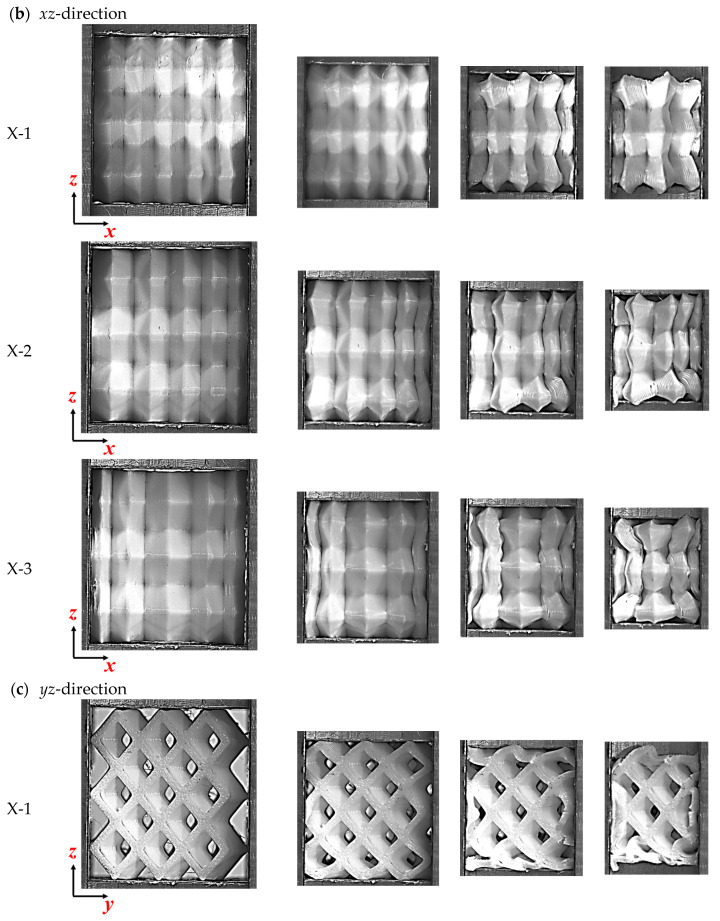
The deformation processes of different models: (**a**) *xy*-direction, (**b**) *xz*-direction, and (**c**) *yz*-direction.

Figure 16 presents the deformation evolution under equibiaxial compression in the *xy*-, *xz*-, and *yz*-directions. The strain shown in the figure refers to the strain in the *x*-direction for the *xy*- and *xz*-directions, and the strain in the *z*-direction for the *yz*-direction. The deformation behavior of the origami units is governed by the local crease rotation and the resulting change in structural stiffness. According to Equation (2), a larger acute angle ϕ corresponds to a larger crease angle α, resulting in higher folding resistance, whereas a smaller acute angle facilitates earlier deformation initiation. Therefore, the regions with smaller ϕ values are more prone to local bending, hinge rotation, and progressive folding under compression. For Model X-2, where the acute angle decreases monotonically from left to right, deformation initiates in the right-hand region and sequentially propagates toward stiffer regions, indicating a progressive collapse mechanism. In contrast, Model X-3 exhibits a symmetric angle-gradient distribution, in which the acute angle increases from both ends toward the middle. As a result, the outer layers with smaller acute angles deform first, followed by progressive collapse of the central region. This deformation pattern indicates that the symmetric gradient promotes a more distributed collapse process and delays the deformation of the stiffer middle layers. Overall, the observed response is dominated by progressive folding, local hinge rotation, and load transfer between neighboring regions, rather than abrupt global collapse. Since all models share the same deformation mode under equibiaxial compression in the *yz*-direction, only the results of Model X-1 are displayed. It is found that the gradient design along the in-plane *x*-direction has no effect on the deformation in the in-plane *y*-direction and out-of-plane *z*-direction, and all layers of all models deform simultaneously in the in-plane *y*-direction and out-of-plane *z*-direction.

#### 3.2.2. Biaxial Stress–Strain Response

**Figure 17:**
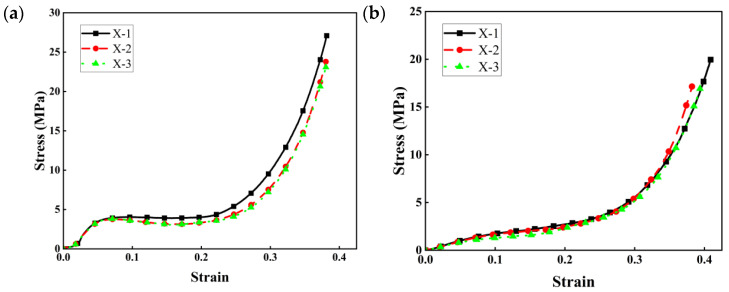
Stress–strain curve under equalbiaxial compression in the *xy*-direction: (**a**) *x*-direction and (**b**) *y*-direction.

**Figure 18:**
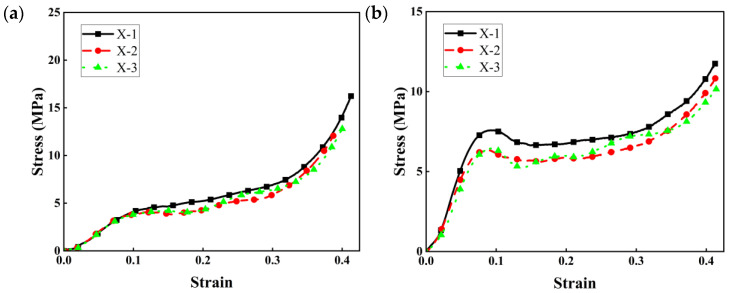
Stress–strain curve under equalbiaxial compression in the *yz*-direction: (**a**) *y*-direction, and (**b**) *z*-direction.

**Figure 19:**
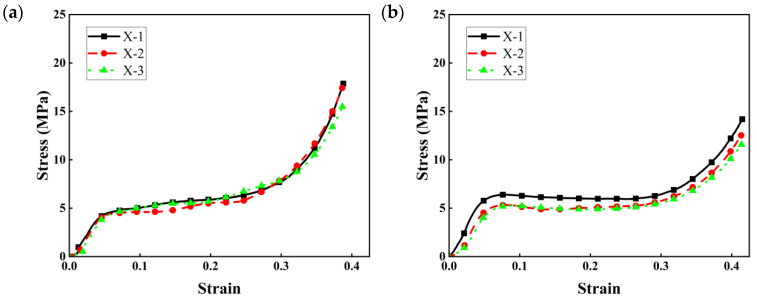
Stress–strain curve under equalbiaxial compression in the *xz*-direction: (**a**) *x*-direction, and (**b**) *z*-direction.

Figure 17, Figure 18 and Figure 19 depict the stress–strain curves under equibiaxial compression in different directions, respectively. It can be observed that the stress–strain curves of all models under equibiaxial compression in various directions can still be roughly divided into three stages: the elastic, plastic, and densification stages. In the elastic stage, for all models under different directional combinations, the elastic modulus in each individual direction of every combination is nearly identical. In this stage, the stress rapidly reaches the yield point at a small strain. Nevertheless, the stress–strain curve obtained from compression along the *y*-direction exhibits a distinct difference compared with those in the other two directions. No obvious peak or plateau force appears in the *y*-direction, and the slope of the stress–strain curve changes slightly between the elastic and plastic stages, indicating an overall continuous upward trend. By contrast, the stress–strain curves in the *x*- and *z*-directions exhibit clear peak and plateau forces.

**Figure 20:**
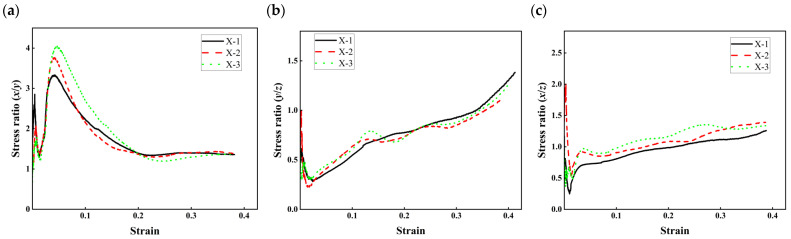
Stress ratio curves under equalbiaxial compression in different directions: (**a**) *xy*-direction, (**b**) *yz*-direction, and (**c**) *xz*-direction.

Figure 20 shows the stress ratio relationships under equibiaxial compression in different directions. In the *xy*-direction, the stress ratios of the three models are consistently greater than 1, indicating that the *x*-direction possesses higher stiffness and carries a larger stress contribution than the *y*-direction during compression. In addition, the amplitude of the stress ratio of the gradient models is higher than that of the uniform model. The stress ratio curves of all models exhibit slight fluctuations at small strains, then rise continuously until the strain reaches approximately 0.05, followed by a decreasing tendency and finally tend to be stable. The peak stress ratio increases from approximately 3.3 for X-1 to about 3.7 and 4.0 for X-2 and X-3, respectively, indicating that the in-plane gradient enhances the early-stage stress imbalance between the x- and y-directions. In the stable stage, the ratios decrease to approximately 1.1–1.3, suggesting that the directional stress difference becomes weaker at larger strains. In the *yz*-direction, all stress ratios remain less than 1, demonstrating that the *z*-direction exhibits greater stiffness during compression. With increasing deformation, the ratios gradually increase and approach or exceed 1 at larger strains, indicating that the stress contribution of the *y*-direction increases during the later stage. The amplitude of the stress ratio of the gradient models is close to that of the uniform model, suggesting that the in-plane gradient has a limited influence on the *y*/*z* stress transfer compared with its effect in the *xy*-direction. The variation trend in the *xz*-direction is similar to that in the *yz*-direction, implying that the constraint of the *z*-direction exerts a stronger enhancement effect on the load-bearing capacity of the *x*-direction than the constraint of the *x*-direction does on the load-bearing capacity of the *z*-direction.

**Figure 21:**
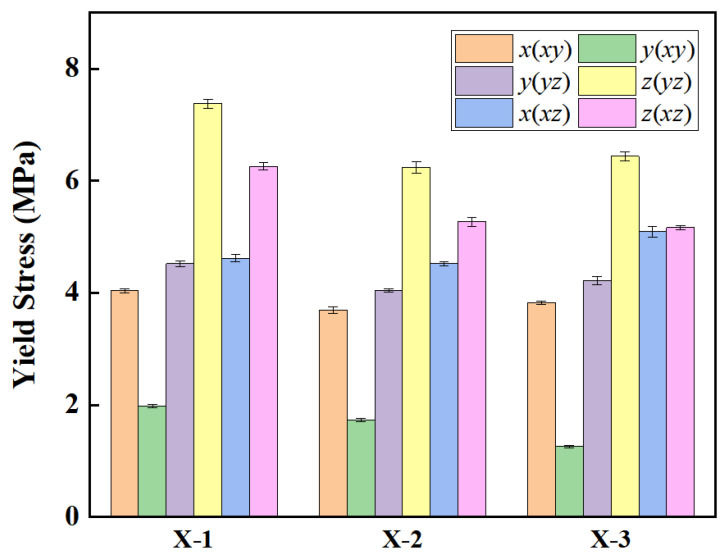
Comparison of yield stress under equalbiaxial compression in different directions.

It can be seen from Figure 21 that under equibiaxial compression in the *xy*-, *yz*- and *xz*- direction, the yield stress of the gradient model is lower than that of the uniform model. Under *xy*-direction compression, the yield stress decreases by 5.4–8.7% in the *x*-direction and by 12.6–36.4% in the *y*-direction. Under *yz*-direction compression, reductions of 6.6–10.4% and 12.7–15.4% are observed in the *y*- and *z*-directions, respectively. Under *xz*-direction compression, the *z*-direction yield stress decreases by 15.8–17.4%, whereas the variation in the *x*-direction remains relatively small. This indicates that the variable-angle gradient design along the in-plane *x*-direction reduces the yield stress of the Miura-ori metamaterial under equibiaxial compression in the *xy*-, *yz*- and *xz*-direction. The largest reduction reaches 36.4%, indicating that the in-plane gradient design generally weakens the initial load-bearing capacity of the structure.

By comparing the yield stress of the same model under equibiaxial compression in different directions, both the uniform model and the gradient model exhibit the maximum overall average yield stress in the *yz*-direction. Comparing the *x*-direction yield stress under equibiaxial compression in the *xy*-direction and *xz*-direction, as well as the *y*-direction yield stress under equibiaxial compression in the *xy*-direction and *yz*-direction, it is found that the yield stresses under equibiaxial compression in the *xz*-direction and *yz*-direction are both higher than those in the *xy*-direction. In terms of the *z*-direction yield stress under equibiaxial compression in the *yz*-direction and *xz*-direction, the value under *yz* equibiaxial compression is greater than that under *xz* equibiaxial compression.

**Figure 22:**
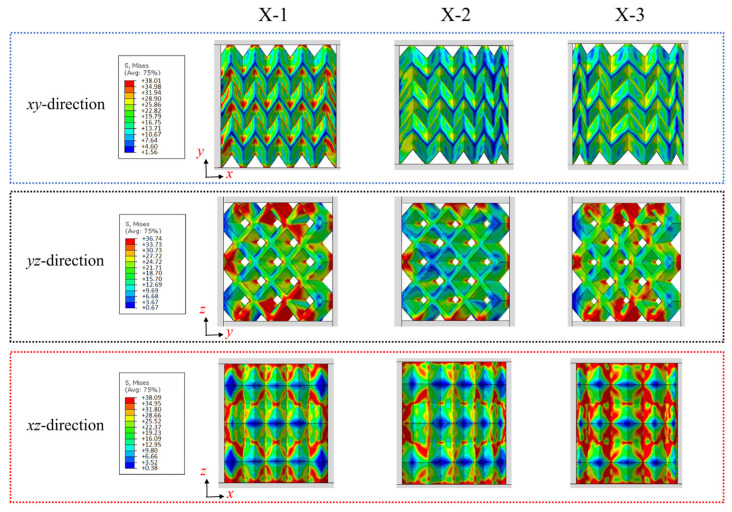
Von Mises stress distributions at the onset of yielding for in-plane gradient Miura-ori structure under equibiaxial compression.

The von Mises stress distributions at the onset of yielding obtained from finite element simulations are presented in Figure 22. Compared with the uniform model (X-1), the gradient models (X-2 and X-3) exhibit lower overall stress levels under *xy*-direction compression, consistent with the experimentally observed reduction in yield stress. Under *yz*-direction compression, X-2 shows a noticeably more non-uniform stress distribution with larger low-stress regions, corresponding to the significant decrease in yield stress. In contrast, the stress distributions under *xz*-direction compression remain relatively similar among the three models, which agrees with the relatively small variation in *x*-direction yield stress. These results indicate that the in-plane gradient design modifies the stress distribution at the onset of yielding, leading to the reduced yield strength observed in the experiments.

**Figure 23:**
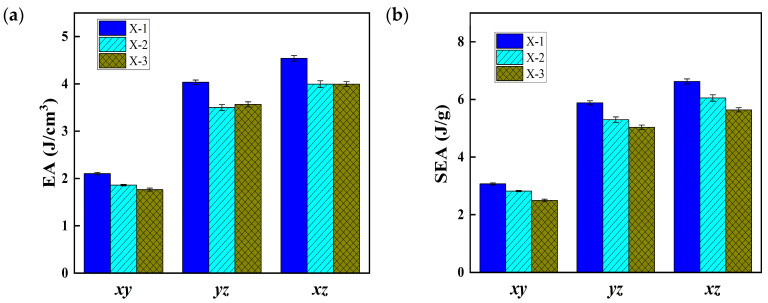
Comparison of EA and SEA for different models under equalbiaxial compression in various directions: (**a**) EA and (**b**) SEA.

The comparison of specific energy absorption among different variable-angle gradient models is presented in Figure 23. It can be observed that, under equibiaxial compression in the *xy*-direction, compared with the uniform model X-1, the EA of the gradient models X-2 and X-3 decreases by 11.54% and 16.04%, respectively, while their SEA decreases by 14.41% and 20.48%, respectively. In the *yz*-direction, relative to the uniform model X-1, the EA of gradient models X-2 and X-3 is reduced by 13.28% and 11.61%, and the corresponding SEA decreases by 16.08% and 16.28%. In the *xz*-direction, compared with model X-1, the EA of gradient models X-2 and X-3 declines by 12.04% and 12.07%, respectively, and their SEA drops by 14.87% and 16.72%, respectively. Previous studies [39] on in-plane gradient Miura-ori structures have shown that, compared with uniform non-gradient configurations, graded structures generally exhibit superior energy absorption performance under uniaxial compression, with the SEA increasing by up to 68% under optimal gradient conditions. However, the present results indicate that under biaxial loading, the in-plane gradient configuration reduces SEA. By comparing the EA values of the same model under equibiaxial compression along different directions, the uniform model and gradient models show a consistent trend, following the order: ${EA}_{xz}>{EA}_{yz}>{EA}_{xy}$.

### 3.3. Engineering Significance of Gradient Design

Although most gradient configurations exhibit a reduction in EA and SEA compared with the uniform structure, this behavior does not necessarily diminish the engineering significance of the proposed designs. Instead, the results indicate that the introduction of geometric gradients primarily serves as a strategy for tailoring deformation modes and tuning the anisotropic mechanical response of Miura-ori metamaterials, rather than maximizing global energy absorption.

The observed reduction in EA and SEA can be attributed to the fact that gradient-induced stiffness heterogeneity promotes earlier localized deformation and reduces the ability of the structure to sustain uniform plastic deformation. However, this localized deformation behavior enables controllable deformation sequences and direction-dependent mechanical responses, which are highly desirable in applications requiring programmable collapse, directional compliance, or controlled load redistribution.

Notably, the out-of-plane gradient configuration under xz loading demonstrates improved energy absorption performance, indicating that the effectiveness of gradient design is strongly dependent on the loading direction. This directional sensitivity suggests that gradient architectures can be used to achieve anisotropic energy management, enabling mechanical performance to be selectively enhanced in specific loading scenarios. Therefore, the proposed gradient designs provide a trade-off between energy absorption capacity and deformation programmability, which is a key design consideration in mechanical metamaterials.

## 4. Conclusions

This study conducted equalbiaxial compression experiments on both uniform and gradient Miura-ori metamaterials. All specimens were fabricated from resin material using 3D printing technology. Experimental investigations were conducted to explore the mechanical properties and deformation behaviors of Miura-ori metamaterials with gradient designs in the out-of-plane direction and in-plane *x*-direction under equiaxial compressive loading along different orientations. The main conclusions are as follows:

(1) The deformation behavior of the gradient structures was strongly governed by the spatial distribution of the acute angle. Under *xz*- and *yz*-direction compression, layers with smaller acute angles deformed preferentially and triggered progressive collapse. In contrast, the uniform structures exhibited nearly synchronous deformation throughout the specimen.

(2) The out-of-plane gradient design significantly affected the yield characteristics of the Miura-ori metamaterials. Under *xy*-direction compression, the yield stress in the x-direction increased by 6.8–14.3%, while that in the y-direction decreased by 29.8–38.3% relative to the uniform model. Under *xz*-direction compression, the *x*-direction yield stress increased by 10.0–11.2%, whereas the *z*-direction yield stress decreased by 3.9–10.3%. Under *yz*-direction compression, the maximum reduction in yield stress reached 19.7%.

(3) The out-of-plane gradient design exhibited direction-dependent energy absorption performance. Compared with the uniform model, the SEA of the gradient structures decreased by 11.9–16.9% under *xy*-direction compression and by 5.7–12.0% under *yz*-direction compression. However, under *xz*-direction compression, the SEA increased by 6.5–8.6%, demonstrating that the out-of-plane gradient configuration is beneficial for energy absorption when loading involves the *z*-direction.

(4) The in-plane gradient design generally weakened the mechanical performance. Compared with the uniform model, the yield stress decreased by up to 36.4%, and the SEA decreased by 14.4–20.5% under *xy*-direction compression, 16.1–16.3% under *yz*-direction compression, and 14.9–16.7% under *xz*-direction compression. These results indicate that introducing an acute-angle gradient along the *x*-direction reduces both the load-bearing capacity and energy absorption capability of the structure.

## Figures and Tables

**Table 1:** Printing parameters for ABS-printed samples.

Parameter	Value
Nozzle diameter (mm)	0.4
Layer height (mm)	0.2
Orientation (°)	0
Platform temperature (°C)	80
Nozzle temperature (°C)	240
Printing speed (mm/s)	50
Infill percentage (%)	100

**Table 2:** Material properties of ABS resin.

Density (g/cm^3^)	Young’s Modulus (MPa)	Poisson’s Ratio	Yield Stress (MPa)
1.2	2172	0.3	6.17

**Table 3:** Unit geometric parameters of uniform Models Z-1 and X-1.

Model	Acute Angle ϕ (°)	$\eta_{A}$ (°)	$\eta_{Z}$ (°)	$L_{x}$ (mm)	$L_{y}$ (mm)	$L_{z}$ (mm)	Cell Wall Thickness (mm)
Z-1	59	81.50	77.37	25.00	24.66	25.00	1
X-1	60	78.46	75.52	24.50	24.03	25.56	1

**Table 4:** Geometric parameters of the gradient models for the out-of-plane z-direction.

Model	The Acute Angle ϕ Measured from Top to Bottom (°)	$L_{x}$ (mm)	$L_{y}$ (mm)	$L_{z}$ (mm)	Cell Wall Thickness (mm)
Z-2	48–52–56–60–64–68	25.00	24.66	25.03	1
Z-3	55–59–63–63–59–55	25.00	24.66	25.33	1

**Table 5:** Geometric parameters of the gradient models for the in-plane x-direction.

Model	The Acute Angle ϕ Measured from Left to Right (°)	$L_{x}$ (mm)	$L_{y}$ (mm)	$L_{z}$ (mm)	Cell Wall Thickness (mm)
X-2	56–58–60–62–64	24.68	24.03	25.56	1
X-3	56–61–66–61–56	24.81	24.03	25.56	1

## Data Availability

The original contributions presented in this study are included in the article. Further inquiries can be directed to the corresponding author.
